# Variational autoencoders learn transferrable representations of metabolomics data

**DOI:** 10.1038/s42003-022-03579-3

**Published:** 2022-06-30

**Authors:** Daniel P. Gomari, Annalise Schweickart, Leandro Cerchietti, Elisabeth Paietta, Hugo Fernandez, Hassen Al-Amin, Karsten Suhre, Jan Krumsiek

**Affiliations:** 1 Institute of Computational Biology, Helmholtz Center Munich—German Research Center for Environmental Health, 85764 Neuherberg, Germany; 2 Technical University of Munich—School of Life Sciences, 85354 Freising, Germany; 3 Department of Genetics, Stanford University School of Medicine, Stanford, CA USA; 4 Department of Physiology and Biophysics, Weill Cornell Medicine, Institute for Computational Biomedicine, Englander Institute for Precision Medicine, New York, NY 10021 USA; 5 Department of Medicine, Hematology and Oncology Division, Weill Cornell Medicine, New York, 10065 NY USA; 6 Albert Einstein College of Medicine-Montefiore Medical Center, Bronx, NY USA; 7 Moffitt Malignant Hematology & Cellular Therapy at Memorial Healthcare System, Pembroke Pines, FL USA; 8 Department of Psychiatry, Weill Cornell Medicine—Qatar, Education City, P.O. Box 24144, Doha, Qatar; 9 Department of Physiology and Biophysics, Weill Cornell Medical College—Qatar Education City, Doha, Qatar

**Keywords:** Machine learning, Metabolomics, Type 2 diabetes, Acute myeloid leukaemia, Schizophrenia

## Abstract

Dimensionality reduction approaches are commonly used for the deconvolution of high-dimensional metabolomics datasets into underlying core metabolic processes. However, current state-of-the-art methods are widely incapable of detecting nonlinearities in metabolomics data. Variational Autoencoders (VAEs) are a deep learning method designed to learn nonlinear latent representations which generalize to unseen data. Here, we trained a VAE on a large-scale metabolomics population cohort of human blood samples consisting of over 4500 individuals. We analyzed the pathway composition of the latent space using a global feature importance score, which demonstrated that latent dimensions represent distinct cellular processes. To demonstrate model generalizability, we generated latent representations of unseen metabolomics datasets on type 2 diabetes, acute myeloid leukemia, and schizophrenia and found significant correlations with clinical patient groups. Notably, the VAE representations showed stronger effects than latent dimensions derived by linear and non-linear principal component analysis. Taken together, we demonstrate that the VAE is a powerful method that learns biologically meaningful, nonlinear, and transferrable latent representations of metabolomics data.

## Abstract (web summary)

Variable autoencoders offer an alternative way to interrogate metabolomic data and identify meaningful, non-linear relationships.

## Introduction

Modern metabolomics experiments yield high-dimensional datasets with hundreds to thousands of measured metabolites in large human studies with thousands of participants^1^. Such datasets are routinely generated to profile the molecular phenotype of disease and identify the underlying pathological mechanisms of action^2–5^. Extracting systemic effects from high-dimensional datasets requires dimensionality reduction approaches to untangle the high number of metabolites into the processes in which they participate. To this end, linear dimensionality reduction methods, such as principal component analysis (PCA) and independent component analysis (ICA), have been extensively applied to high-dimensional biological data^6–10^. However, metabolic systems, like most complex biological processes, contain non-linear effects which arise due to high-order enzyme kinetics and upstream gene regulatory processes^11,12^. For example, metabolite ratios are an intuitive and widely used approach to detect non-linear effects in metabolomics data, approximating the steady state between reactants and products of metabolic reactions^13,14^. Extending this concept, systematic methods that take nonlinearities into account are required to correctly recover the functional interplay between metabolites in an unbiased fashion.

Autoencoders (AEs) are a type of neural network architecture developed as an unsupervised dimensionality reduction method that can capture non-linear effects^15^. AEs reduce high-dimensional data into latent variables through an encoding/decoding process which recreates the input data after passing through a lower dimensional space. Once the model is fitted, the latent variables represent a compact, often easier-to-interpret version of the original data. While AEs have been successfully used for prediction tasks on biological datasets^16^, they tend to learn latent spaces specifically fitted to the input dataset and are therefore not generalizable to biological phenomena absent from the training data^17^. To address this, Variational Autoencoders (VAEs) were introduced as a probabilistic extension of the AE architecture that constrains the latent variables to follow a predefined distribution^18^. With this extension, the VAE not only reconstructs the input data, but infers the generative process behind the data, leading to high generalizability across datasets. The VAE architecture has, for example, proven effective for predicting cell-level response to infection from transcriptomic data in cell types not used during training, and predicting drug response from gene expression data where drug response information was sparse^17,19,20^. In addition, latent dimensions of VAE models trained on gene expression data have been shown to strongly associate with clinical parameters^21^ and have outperformed other common low-dimensional representation methods (PCA and k-means clustering) in capturing biologically meaningful pathways^22^.

The application of deep learning architectures to metabolomics datasets has considerably lagged behind most other omics^23^ due to the unavailability of large metabolomics cohorts. One previous study used (non-variational) AEs on a small set of metabolomics samples (*n* = 271), demonstrating superior predictability of ER status in breast cancer patients using the latent representations of the model over common machine learning methods^24^. By applying VAE architectures to large-scale metabolomics data, we have the potential to learn more accurate latent dimension representations that systematically account for non-linear effects. In addition, the probabilistic structure of VAEs will learn latent dimensions that are generalizable across multiple datasets.

**Fig. 1:**
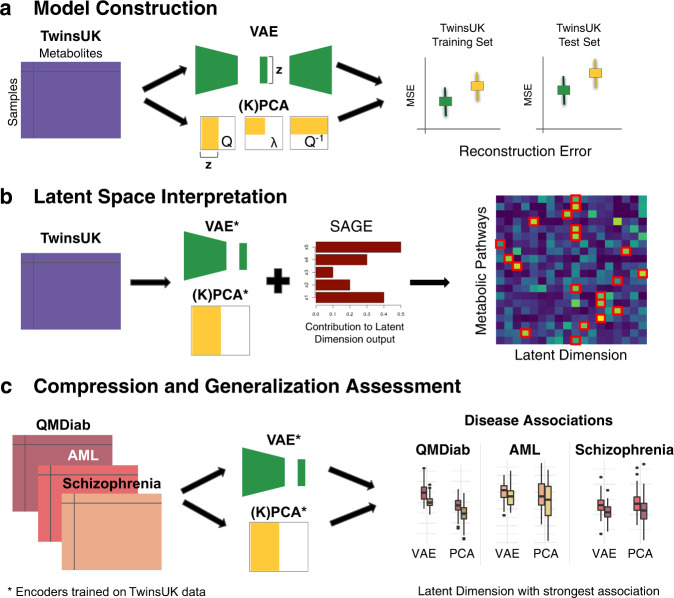
Overview of our approach. **a** VAE, linear PCA, and non-linear kernel (K)PCA models were trained using training and test sets in the TwinsUK dataset (*n* = 4644 samples, *p* = 217 metabolites). Model performance was then evaluated using Mean Squared Error (MSE) of metabolite correlation matrix reconstruction. **b** The SAGE method was applied to calculate the contribution of individual metabolites, sub-pathways and super-pathways to each latent dimension. **c** QMDiab (*n* = 358), AML (*n* = 85), and Schizophrenia (*n* = 201) datasets were encoded using VAE and PCA models trained on the TwinsUK data. Latent dimensions of each model were then associated with disease phenotypes.

In this paper, we trained a VAE model on 217 metabolite measurements in 4644 blood samples from the TwinsUK study^25^ and evaluated our model performance in comparison to linear and non-linear PCA models (Fig. 1a). To investigate the biological relevance of the learned VAE and PCA latent dimensions, we employed the Shapley Additive Global Importance (SAGE) method^26^, which determines the contribution of each input to each latent dimension. We calculated SAGE values at different granularities, i.e., metabolites, *sub-pathways*, and *super-pathways* (Fig. 1b). We then applied the models on three additional blood metabolomics datasets to test their ability to recover disease phenotypes in unseen datasets: Type 2 Diabetes diagnosis in The Qatar Metabolomics Study on Diabetes (QMDiab, *n* = 358), therapy response in an acute myeloid leukemia dataset (AML, *n* = 85), and schizophrenia diagnosis in a third validation dataset (*n* = 201) (Fig. 1c).

## Results

### VAE model construction and fitting

**Fig. 2:**
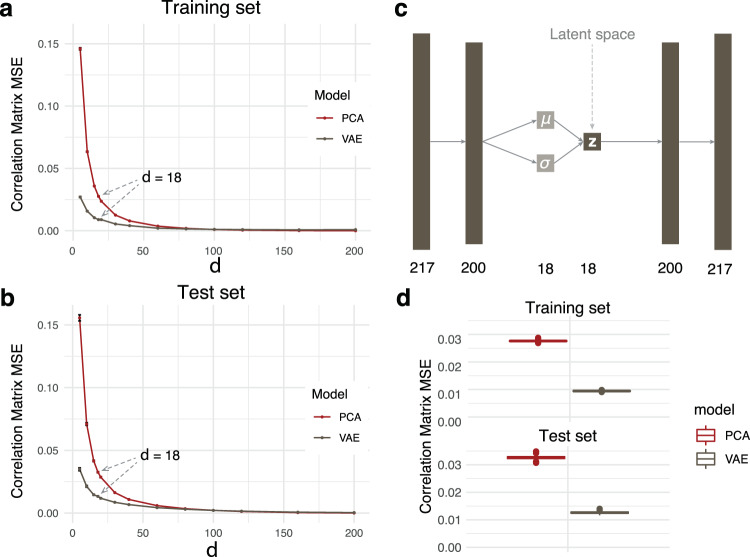
VAE and PCA model construction on the TwinsUK dataset. **a** Training and **b** test set metabolite correlation matrix reconstruction for a range of latent dimensionality values *d*. The slope of the VAE curve plateaued after *d* = 18. Error bars correspond to one standard deviation from bootstrapping (*n* = 1000 iterations). **c** Final VAE architecture, where *μ* is the mean vector and σ is the standard deviation vector that generates the latent space **z**. **d** Reconstruction MSE for latent dimensionality *d* = 18 on training and test sets. The box represents the interquartile range (IQR), whiskers are up to 1.5x IQR, and plotted points are outliers. The VAE preserved feature correlations substantially better than PCA. Kernel PCA-based results can be found in Supplementary Figs 3 and 4.

Our VAE architecture consisted of an input/output layer, an intermediate layer and a latent layer. We split the TwinsUK cohort into an 85% training and a 15% test set, and the training set was used to optimize the hyperparameters in the VAE model. Keras Tuner^27^ identified the following optimal hyperparameters: Intermediate layer dimensionality = 200, learning rate = 0.001, and Kullback-Leibler (KL) divergence weight = 0.01 (see Methods). With these parameters fixed, we optimized the dimensionality *d* of the latent layer **z** by calculating the reconstruction MSE of the correlation matrix (CM-MSE) of metabolites (Fig. 2a, b). We observed that the CM-MSE curve plateaus after *d* = 18, indicating that increasing the latent dimensionality beyond this value only marginally improved the models. The final architecture of the model consisted of a 217-dimensional input/output layer (the number of metabolites in our datasets), a 200-dimensional intermediate layer, and an 18-dimensional latent layer (Fig. 2c).

We used principal component analysis (PCA) as a baseline model to compare the VAE to a linear latent variable embedding method. To this end, we fitted a PCA on the TwinsUK train data and extracted the first *d* = 18 dimensions, i.e., principal components. To further compare our VAE results to a non-linear latent variable method, we used the same fit and extraction procedures using kernel (K)PCA models with cosine similarity, sigmoid, RBF, and polynomial kernel transformations. On training data, neither PCA nor VAE outperformed KPCA data matrix reconstruction. On testing data, however, both PCA and VAE outperformed the KPCA models, with PCA showing better reconstruction accuracy of the data matrix than the VAE (Supplementary Figs 1 and 2). All non-linear models, KPCA and VAE, outperformed PCA in correlation matrix reconstruction via CM-MSE in both the TwinsUK train and test set (Fig. 2d, Supplementary Fig. 3).

This difference between the MSE on the correlation matrix and the more commonly used sample-wise MSE^18^ outlines ambiguities in the methods to assess reconstruction performances. Notably, other authors have shown previously that sample reconstruction performance does not necessarily imply better model performance^19,20^, as evidenced by the large discrepancy of KPCA sample reconstruction between training and test sets. However, our results suggest that while the non-linear methods do not reconstruct the original data matrix precisely, they are superior to PCA at preserving metabolite correlations.

### Interpretation of VAE latent space dimensions in the context of metabolites and pathways

**Fig. 3:**
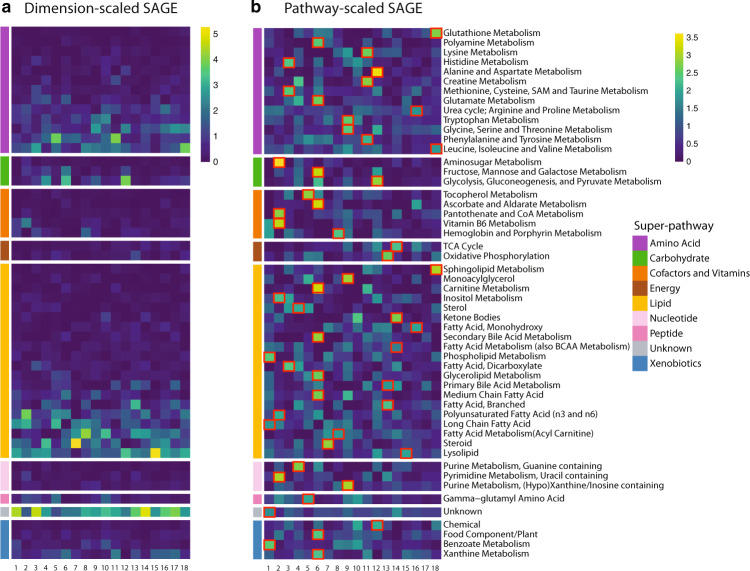
Sub-pathway-level SAGE values for the VAE latent dimensions. **a** SAGE values were scaled by dimension, i.e., set to standard deviation 1 for each column in the matrix. This highlights pathways that contributed the most to each dimension. Lipid and amino acid super-pathways showed the highest values for most dimensions, which can be attributed to the high number of metabolites in those pathways. **b** SAGE values were scaled by pathway, i.e., set to standard deviation 1 for each row in the matrix. This highlights dimensions that contributed to a pathway the most. Taking into consideration the largest scaled SAGE values per pathway (red square marks), almost all sub-pathways were represented by unique dimensions. The combination of these key sub-pathways of a dimension outlined the distinct cellular mechanisms a dimension encodes.

We evaluated the composition of all latent dimensions in the context of metabolic pathways. For each metabolite in our dataset, a “sub-pathway” and “super-pathway” annotation was available (see Methods). Sub-pathways refer to biochemical processes such as “TCA Cycle” and “Sphingolipid Metabolism”, while super-pathways are broad groups such as “Lipid” and “Amino acid”. To provide insights into the processes represented by different VAE dimensions, we computed SAGE scores^26^, a measure of model feature relevance, at the level of metabolites, sub-pathways and super-pathways (Fig. 3 and Supplementary Fig. 4). PCA and KPCA results can be found in Supplementary Figs 5–9.

The VAE sub-pathway heatmap (Fig. 3a) shows that nearly all dimensions have major contributions by lipid and amino acid super-pathways. The prevalence of the two super-pathways can be attributed to the fact that those groups contain the largest number of metabolites in the dataset. Note that we deliberately omitted the “Unknown” molecule group, which refers to unidentified metabolites that could originate from any pathway.

Inspecting the SAGE values in the other direction, almost all sub-pathways are predominantly represented by a single VAE dimension that captures the respective pathway the most (Fig. 3b, red square marks). For instance, “glycolysis, gluconeogenesis and pyruvate metabolism” is functionally related to the sub-pathway pathway “Alanine and Aspartate Metabolism” through the glucose-alanine cycle^28^, and both were represented by VAE dimension 12. Another interesting example is VAE dimension 14, which captured essential catabolic processes, such as ketone bodies, fatty acid metabolism, and the TCA cycle. Taken together, these results show that VAE latent dimensions capture a complex mix of functionally related sub-pathways, thus capturing major metabolic processes in the dataset.

In contrast, PCA and KPCA dimensions 1 to 5, which by construction represent the highest variations in the data, nonspecifically captured various sub-pathways. Most other PCA and KPCA dimensions also primarily contained unrelated sub-pathways (Supplementary Figs 5–9).

### VAE latent space captures signals in unseen diabetes, schizophrenia, and cancer metabolomics datasets

**Fig. 4:**
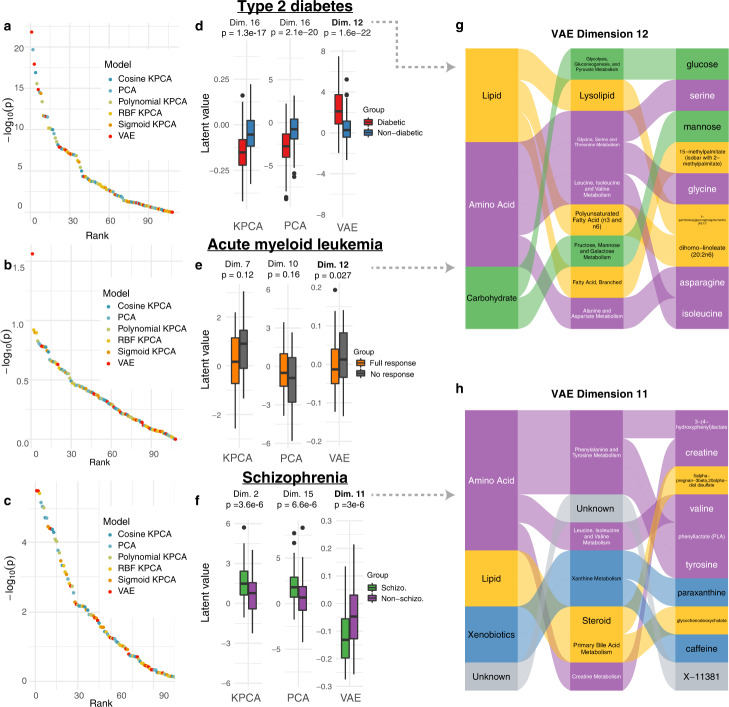
VAE latent space associations with clinical outcomes. **a**–**c** Sorted −log_10_(*p*-value) for all VAE, PCA and KPCA dimensions for the type 2 diabetes (*n* = 358), AML (*n* = 85), and schizophrenia (*n* = 201) datasets, respectively. The highest scoring VAE dimensions showed lower *p*-values than the highest scoring KPCA dimensions, and considerably lower *p*-values than the highest scoring PCA dimensions for all datasets. **d**–**f** Boxplots of latent dimension values for latent space dimensions with the lowest *p*-values for the three datasets. The box represents the interquartile range (IQR), whiskers are up to 1.5x IQR, and plotted points are outliers. **g** Contributions of super-pathways, sub-pathways and metabolites determined by SAGE values to dimension 12, the highest scoring VAE latent dimension for QMDiab and AML. This dimension was driven by lipids, amino acids, and carbohydrates. **h** Contributions of super-pathways, sub-pathways and metabolites determined by SAGE values to dimension 11, the highest scoring VAE latent dimension for Schizophrenia. This dimension was largely driven by amino acid metabolism. *p* = *p*-value. Schizo. = schizophrenic.

We investigated whether VAE latent dimensions learned on the TwinsUK data contained information that is generalizable to other datasets. To this end, we encoded metabolomics data from three clinical datasets, type 2 diabetes, acute myeloid leukemia (AML), and schizophrenia using the VAE, PCA, and KPCA encoders trained on the TwinsUK dataset. For each VAE, PCA, and KPCA latent dimension, we performed a two-sided *t*-test between diabetic vs. non-diabetic individuals, full vs. no response in an AML clinical trial, and schizophrenic vs. non-schizophrenic individuals, respectively. Across all datasets, the best performing VAE dimensions associated substantially stronger with the patient groups than any of the PCA or KPCA dimensions (Fig. 4a–f). The strength of associations between VAE dimensions and disease parameters were comparable to single metabolite associations (Supplementary Data 1 and 2). However, unlike the VAE dimensions, these univariate associations do not represent system-level mechanisms related to the diseases. Notably, results did not differ substantially for different dimensionalities than *d* = 18 (Supplementary Fig. 15).

To obtain a better understanding of the driving factors of the VAE associations, we ranked pathways and metabolites by their calculated SAGE values (Fig. 4g–i):

#### Type 2 diabetes

VAE latent dimension 12 showed the highest association with type 2 diabetes, with a considerably stronger signal than the second highest association by PCA dimension 16 (*p* = 1.6 × 10^−22^ vs. *p* = 2.1 × 10^−20^, respectively; Fig. 4d). The top sub-pathways were “glycolysis, gluconeogenesis, and pyruvate metabolism”, “Lysolipid”, and “Glycine, Serine, and Threonine metabolism”. The top-ranking metabolite in dimension 12 was glucose, which is a main factor directly affected by the disease, and thus served as a positive control. Other high-ranking metabolites included serine, mannose, and glycine (Fig. 4g). Reduced levels of the glucogenic amino acids serine and glycine have repeatedly been reported in Type 2 Diabetes cases as impaired glucose uptake in insulin-resistant cells triggers hepatic gluconeogenesis, consuming these amino acids^29–31^. Furthermore, plasma levels of mannose have also been found to associate with type 2 diabetes diagnosis^32^. We then correlated dimension 12 with clinical lab measurements from the QMDiab study and found a strong association between this dimension and HbA1c, a widely used diabetes biomarker^33,34^ (*p* = 6.2 × 10^−45^ compared to PCA *p* = 1.1 × 10^−30^, and the strongest associated KPCA dimension *p* = 6.0 × 10^−38^, Supplementary Figs 10–12). This finding demonstrates how a quantitative disease biomarker can carry more information than a crude disease yes/no classification, and further highlights the higher information content in the VAE latent dimensions compared to PCA.

#### Acute myeloid leukemia (AML)

AML response groups associated an order of magnitude stronger with VAE dimension 12 than with RBF KPCA dimension 10 (*p* = 0.027 vs. *p* = 0.12, respectively; Fig. 4f). Note that the *p*-value would not withstand multiple testing correction (Supplementary Data 1); the detected signal is thus merely suggestive and requires replication in future studies. In addition to the glycolytic signals described above, dimension 12 was also driven largely by the “Lipid” super-pathway, with the “Lysolipid” sub-pathway ranking highly for this dimension. The lysolipid sub-pathway has prominent contributions from the metabolite 1-palmitoleyolglycerophosphocholine, belonging to the glycerophosphocholine (GPC) family, which are products of cellular membrane breakdown^35^. In the clinical trial from which the AML data was taken, the clinical arms were given varying doses of the drug daunorubicin, an anthracycline antibiotic whose mechanism of action includes targeting the cell membrane^36–39^. In fact, the localization of daunorubicin in cellular membranes depends on the daunorubicin/lipid ratio, and therefore differential lipid concentrations between patients could affect drug response^40,41^. In addition, the sub-pathway “Polyunsaturated Fatty Acid (n3 and n6)” was another lipid-related driver of dimension 12. The upregulation of polyunsaturated fatty acids (PUFA) is a trait of anthracyclin treatment^42^, indicating that differential abundance of PUFA may reflect treatment efficacy and thus response in the clinical trial arms. We furthermore investigated correlations of the latent dimensions with 21 major AML-related mutations; the analysis revealed only one significant result, with the *NPM1* mutation associating significantly with polynomial KPCA model dimension 11 (*t*-test, *p* < 0.05, Supplementary Figs 13 and 14). However, this genetic association did not translate to the clinical association with drug response.

#### Schizophrenia

VAE dimension 11 had a stronger association with schizophrenia than the second-ranked RBF KPCA dimension 2 (*p* = 3.0 × 10^−6^ vs. *p* = 3.6 × 10^−6^, respectively; Fig. 4e). The top scoring super-pathway for this dimension (Fig. 4h) was amino acids, with the Phenylalanine and Tyrosine metabolism sub-pathway and its associated metabolites 3-(4-hydroxyphenyl)lactate, phenyllactate (PLA), and tyrosine being highly ranked in this dimension. Phenylalanine and tyrosine are the amino acid precursors for the synthesis of the main neurotransmitter implicated in the neurobiology of schizophrenia, dopamine^43^. Many studies have shown altered transport of tyrosine across the blood-brain barrier as a prominent mechanism in schizophrenic patients^44–46^. In addition, inflammation, increasingly implicated in schizophrenic patients, reduces the activity of the enzyme responsible for converting phenylalanine to tyrosine, such that phenylalanine is instead converted to phenylpyruvic acid, a precursor metabolite to PLA^43,47^. Finally, dimension 11 was strongly driven by the amino acid creatine. Studies have shown that schizophrenia patients have significantly reduced brain levels of creatine compared with healthy controls^48^, and creatine-producing enzymes in serum have long been implicated in schizophrenic psychosis^49,50^.

Taken together, these results suggest that our VAE has learned representations of metabolic processes that are essential in unseen clinical outcomes.

## Discussion

In this study, we trained a VAE on metabolomics data from the TwinsUK population cohort and applied the learned latent representations on unseen data. Notably, non-linear dimensionality reduction methods, including kernel PCA and our VAE model, outperformed the linear PCA model in metabolite correlation matrix reconstruction, underlining the importance of non-linear relationships in metabolomics data. Interpretation of VAE latent dimensions at the metabolite, sub-pathway, and super-pathway level revealed that these dimensions represent functionally related and distinct cellular processes. Moreover, VAE latent dimensions showed substantially stronger disease associations than PCA and even the non-linear KPCA models in unseen Type 2 Diabetes, AML, and schizophrenia datasets. This demonstrates that the VAE learned a latent representation of metabolomics data that is biologically informative and transferable across different cohorts.

The generalizability of the VAE across different datasets is especially remarkable given the vastly different underlying populations of the datasets analyzed here. The VAE was trained on the TwinsUK population cohort, a European-ancestry population cohort consisting predominantly of British women (~92%), while the validation datasets are mixed-gender and multi-ethnic cohorts from the US and Qatar. Despite the existence of these variations in our datasets, our VAE learned a generalized representation of metabolomics data which was able to identify disease-related differences.

The main limitation of our study is the size of the TwinsUK training dataset with *n* = 4644. This is a general issue with human subject metabolomics studies, where even the largest cohorts reach only about *n* = 15,000^51^. Deep learning models are currently more popular in larger datasets of *n* = 60,000 samples or more, such as single cell transcriptomic^52–55^, image^56–58^, and text sources^59^. Learning the variation in such large datasets allows these models to significantly outperform their linear counterparts. Large metabolomics datasets, such as that of the UK BioBank with a sample size of up to *n* = 500,000^60^, will be available in the near future, and will enable the creation of more expressive and deeper VAE models.

To the best of our knowledge, this is the first study to construct a universal latent representation of large-scale metabolomics data using VAEs. Our results show that VAEs are well-suited for metabolomics data analysis and can potentially replace dimensionality reduction approaches, such as PCA, in creating a universal, systems-level understanding of metabolism.

## Methods

### Datasets

The TwinsUK registry is a population-based study of around 12,000 volunteer twins from all over the United Kingdom. The participants have been recruited since 1992 and are predominantly female, ranging in age from 18 to 103 years old^25^. For our study, we included data from 4644 twins (4256 females, 388 males), the subset of TwinsUK for which plasma metabolomics measurements were available. Ethical approval was granted by the St Thomas’ Hospital ethics committee and all participants provided informed written consent.

The QMDiab study was conducted between February and June of 2012 at the Dermatology Department of Hamad Medical Corporation (HMC) in Doha, Qatar. The study population was between the ages of 23 and 71, predominantly of Arab, South Asian, and Filipino descent^61^. For this study, we included plasma data of 358 subjects (176 females, 182 males; 188 diabetic, 177 non-diabetic). The study was approved by the Institutional Review Boards of HMC and Weill Cornell Medicine-Qatar (WCM-Q). Written informed consent was obtained from all participants.

The cohort of patients with acute myeloid leukemia (AML) comes from the ECOG-ACRIN Cancer Research Group phase 3 trial NCT00049517. This study was conducted between December 2002 and November 2008, recruiting 657 patients with AML between the ages of 17 and 60. A subset of these patients had follow-up profiling to determine their response to therapy. For this study, we included the serum metabolomics measurements of 85 subjects of which 43 responded to therapy and 42 did not (34 females, 51 males). The study was approved by the institutional review board at the National Cancer Institute and each of the study centers, and written informed consent was provided by all patients.

For the schizophrenia analysis, metabolomics samples were taken from an antipsychotics study conducted in Qatar between December 2012 and June 2014^62^. A total of 226 participants between the ages of 18 and 65 years of age were recruited, predominantly of Arab descent. For our study, we included plasma metabolomics measurements from 201 subjects (76 females, 125 males; 97 schizophrenic, 104 non-schizophrenic). Approval for the study was obtained from the HMC and WCM-Q Institutional Review Boards, and all participants provided written informed consent.

### Metabolomics measurements and metabolite annotations

Metabolic profiling for all four cohorts was performed using non-targeted ultrahigh-performance liquid chromatography and gas chromatography separation, coupled with mass spectrometry on the Metabolon Inc. platform as previously described^63^. Notably, the AML dataset was based on serum samples, while TwinsUK, QMDiab, and schizophrenia metabolomics were run on plasma samples. However, previous studies have shown that these two sample types are comparable, as shown by high correlations and good reproducibility between plasma and serum measurements in the same blood sample^64^.

For each metabolite measured on the Metabolon platform, a super-pathway and sub-pathway annotation was provided. For super-pathways, we have nine annotations referring to broad biochemical classes, namely “Amino acid”, “Carbohydrate”, “Cofactors and vitamins”, “Energy”, “Lipid”, “Nucleotide”, “Peptide”, “Xenobiotics”, and “Unknown”. Note that “Unknown” is assigned to unidentified metabolites. Furthermore, we have 54 sub-pathway which represent more functional metabolic processes, such as “Carnitine metabolism”, “TCA Cycle”, and “Phenylalanine and Tyrosine Metabolism”.

### Data processing and normalization across datasets

For each dataset, metabolite levels were scaled by their cohort medians, quotient normalized^58^ and then log-transformed. Samples with more than 30% missing metabolites and metabolites with more than 10% missing samples were removed. Missing values were imputed using a k-nearest neighbors imputation method^65^. Datasets with BMI measurements (Schizophrenia, QMDiab, and Twins) were corrected for that confounder and then mean-scaled. 217 metabolites were overlapping between the 4 datasets and were kept for further analysis.

Semi-quantitative, non-targeted metabolomics measurements are inherently challenging to compare across datasets due to heterogeneity between studies. This prevents any machine learning model from being directly transferable from one study to the other. Thus, to ensure comparability, datasets were normalized using a uniform group of participants as a reference set. This group was selected as follows: Male, within a 20-year age range (30–50 for TwinsUK, QMDiab, and schizophrenia, 40–60 for AML due to low sample size of younger participants), BMI between 25 and 30 (not available for AML data, thus not filtered for that dataset), and in the respective control group. Each metabolite in each dataset was then scaled by the mean and standard deviation of their respective uniform sample groups. The assumption of this approach is that the uniform group of reference participants has the same distributions of metabolite concentrations.

### Variational autoencoder fitting

To train our VAE model, we first split the TwinsUK data into 85% training and 15% test sets. We then fixed our VAE architecture to be composed of an input/output layer, an intermediate layer which contains non-linear activation functions, and a *d*-dimensional latent layer. The latent layer consists of a mean vector *μ* and a standard deviation vector *σ*, both of length *d*, which parametrize each latent dimension as a Gaussian probability distribution. This latent space, denoted by ***z***, is constructed by the simultaneous learning of the *μ* and *σ* encoder through the use of a reparameterization trick that enables back propagation during training^18^. The *d* x *d* covariance matrix ∑ of the underlying multivariate Gaussian is assumed to be diagonal (i.e., no correlation across latent dimensions), allowing the covariance matrix to be represented by a single vector *σ* of length d.

For the parameter fitting procedures, all weights were initialized using Keras’ default model weight initialization, i.e., Glorot uniform^66^. Leaky rectified linear units (ReLUs)^67^ were used for non-linear activation functions. The VAE models were trained for 1000 epochs using MSE loss for sample reconstruction and a batch size of 32.

To select the latent dimensionality *d* of our VAE model, we initially fixed this value to *d* = 50. We then optimized the model hyperparameters using Keras Tuner^27^ and the TwinsUK training set with MSE as the objective metric to minimize. We identified the following optimized values: Intermediate layer dimensionality = 200, learning rate = 0.001, and Kullback-Leibler (KL) divergence weight = 0.01. Note that despite our hyperparameter choices, other optimal hyperparameters exist and could be chosen through Keras Tuner. Using these hyperparameters, we then optimized *d* by calculating the reconstruction MSE of the correlation matrix (CM-MSE) of metabolites for *d* = 5, 10, 15, 18, 20, 30, 40, 60, 80, 100, 120, 160, and 200 on the TwinsUK test set. Our final model consisted of a 217-dimensional input/output layer (the number of metabolites in our datasets), a 200-dimensional intermediate layer, and an 18-dimensional latent layer. For all sample encodings in the study, we used their respective *μ* values.

We further performed sensitivity analysis to determine the effect of the choice of *d* on clinical parameter association. We trained 100 models using the architecture above, varying the latent layer dimensionality for *d* = 10, 13, 15, 16, 17, 18, and 20. For each model, we extracted the *p*-value of the highest associated latent dimension, creating a *p*-value distribution for each *d*, which was then compared to the PCA and KPCA associations (Supplementary Fig. 15).

All models were computed on a deep learning-specific virtual machine running on Google Compute Engine with two NVIDIA Tesla K80 GPU dies and 10 virtual CPUs.

### PCA and kernel PCA embedding and reconstructions

We used PCA with *d* = 18 latent dimensions as a baseline model. On the mean-centered TwinsUK training set data matrix with *n* = 3947 samples (rows) and *k* = 217 metabolites (columns), we calculated the rotation matrix ***Q***, a *k* x *k* matrix of eigenvectors ordered by decreasing magnitudes of eigenvalues. To embed a new *m* x *k* dataset ***X*** with *m* samples into the *m* x *d* PCA latent space ***A***, we first calculated ***XQ*** = ***A*** and subsetted to the first *d* columns, denoted by ***A***_*∗, d*_. To simulate the process of encoding and decoding in PCA for dataset ***X***, we calculated the reconstructed dataset as

1 $${\hat{\boldsymbol X}}={\boldsymbol A}_{\ast},{}_{d}{{\boldsymbol Q}^{-1}}_{d,\ast}$$

We used kernel PCA with *d* = 18 latent dimensions as comparative non-linear models. Using the Scikit-learn python package^68^, we applied the four available non-linear kernels to the mean-centered TwinsUK training set data matrix: cosine similarity, sigmoid, radial basis function (RBF), an d polynomial transformations. KPCA hyperparameters were chosen for each individual kernel using the grid search method based on their ability to minimize model MSE. Once transformed, the decomposition, embedding, and encoding/decoding processes were performed identically to those of linear PCA.

### Model assessments

We assessed our KPCA, PCA and VAE models using sample reconstruction mean squared error (MSE) and metabolite-wise correlation matrix MSE (CM-MSE). We calculated CM-MSE by first computing the metabolite-wise correlation matrix of an input dataset and reconstructed input dataset. Afterwards, we calculated the MSE between the upper triangular matrix of the two symmetric correlation matrices.

To calculate a confidence interval for both MSE and CM-MSE between our input and reconstructed data, we randomly sampled the same samples with replacement from the two datasets and then calculated MSE and CM-MSE. We performed this for 1000 iterations.

### Model interpretation

In order to interpret each latent dimension for our VAE, PCA, and KPCA models, we calculated Shapley Additive Global Importance (SAGE) values^26^ for metabolites, sub-pathways, and super-pathways. As contributions of metabolites, sub-pathways, and super-pathways to latent dimensions can vary between multiple trained VAE models, this analysis was performed on a VAE model with average performance from the clinical association analysis. Briefly, SAGE is a model-agnostic method that quantifies the predictive power of each feature in a model while accounting for interactions between features. This is achieved by quantifying the decrease in model performance when combinations of model variables are removed. Since there are exponentially many combinations of variables, a common approach is to sample the feature combination space sufficiently. For each of the tested combinations, a loss function, such as MSE, is used to quantify the decrease in performance compared to the model output (here each VAE, PCA, and KPCA latent dimension) computed using the full model. Then, the mean of all MSEs is calculated, which represents the contribution of the model variables to a latent dimension. To calculate pathway-level SAGE values, metabolites were grouped into pathways and each pathway was treated as a single variable. For each of our VAE, PCA, and KPCA models, we ran SAGE using our TwinsUK test set with default parameters, e.g., marginal sampling size of 512, as suggested by Covert, et al. ^26^. We used the SAGE code from https://github.com/iancovert/sage.

### Reporting summary

Further information on research design is available in the Nature Research Reporting Summary linked to this article.

## Supplementary information

Supplementary Information

Description of Additional Supplementary Files

Supplementary Data 1

Supplementary Data 2

Reporting Summary

## Data Availability

Type 2 diabetes (QMDiab), acute myeloid leukemia (AML), and schizophrenia datasets used in this study are deposited at (https://figshare.com/s/6716415ce4b4e8295f5b). The TwinsUK dataset can be accessed via https://twinsuk.ac.uk/resources-for-researchers/access-our-data/ upon request.

## Code availability

Codes used in this study are available at the GitHub repository https://github.com/krumsieklab/mtVAE and 10.5281/zenodo.6533078^69^.
