# Supplementary material for: Variational autoencoders learn transferrable representations of metabolomics data
Source: Commun Biol. 2022 Jun 30;5:645. doi: 10.1038/s42003-022-03579-3 (PMC9246987; doi:10.1038/s42003-022-03579-3)
Supplement: Supplementary file 1 — Supplementary Information [file 42003_2022_3579_MOESM1_ESM.pdf]

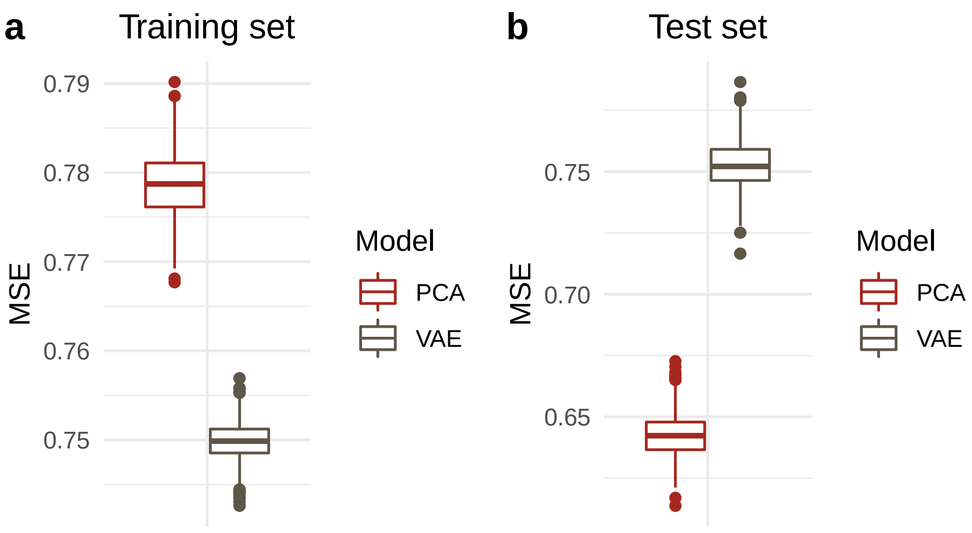

Supplementary Figure 1. Sample reconstruction MSE. a, TwinsUK training and, **b**, test set sample reconstruction MSE for latent dimensionality *d* = 18. The box represents the interquartile range (IQR), whiskers are up to 1.5x IQR, and plotted points are outliers. VAE had a lower reconstruction error in the training set. However, PCA had a lower reconstruction MSE in the training set, implying that PCA performed better at sample reconstruction.

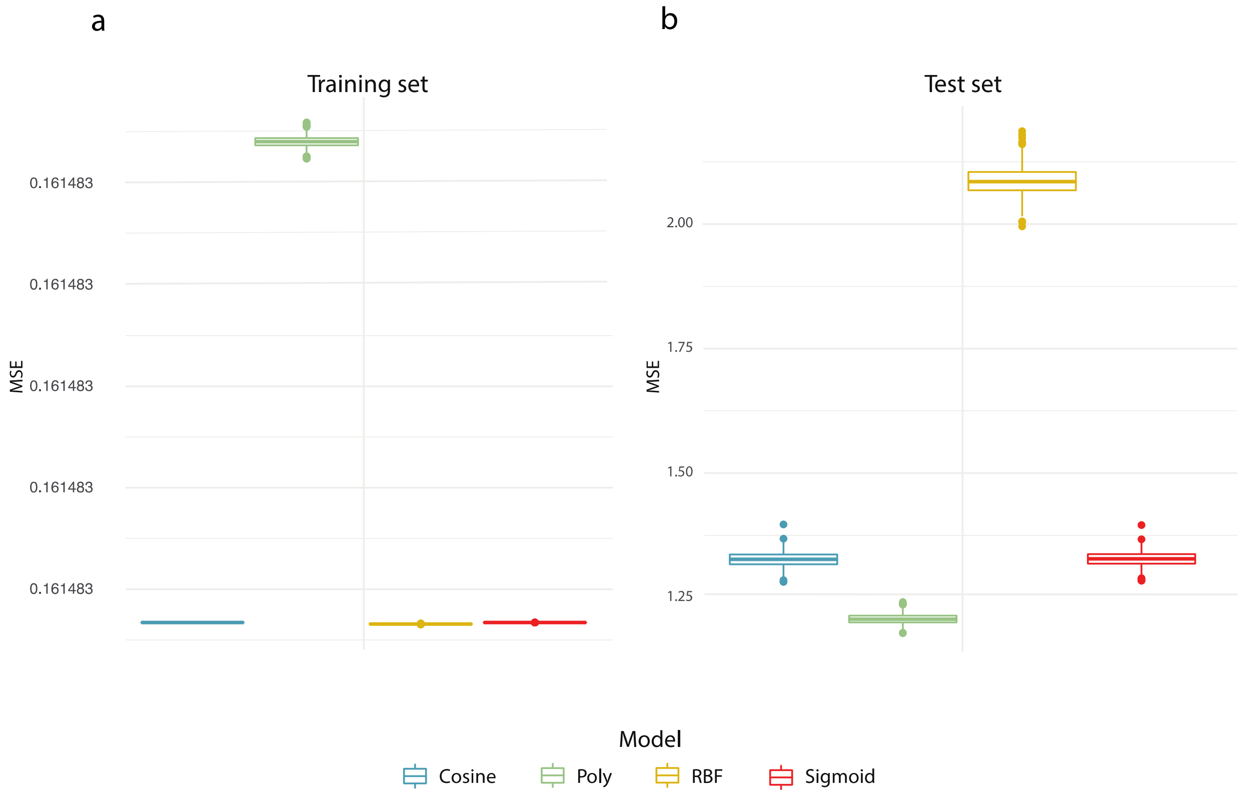

Supplementary Figure 2. KPCA reconstruction MSE. a, KPCA reconstruction sample-wise MSE for latent dimensionality *d* = 18 on training and **b,** test sets. The box represents the interquartile range (IQR), whiskers are up to 1.5x IQR, and plotted points are outliers.

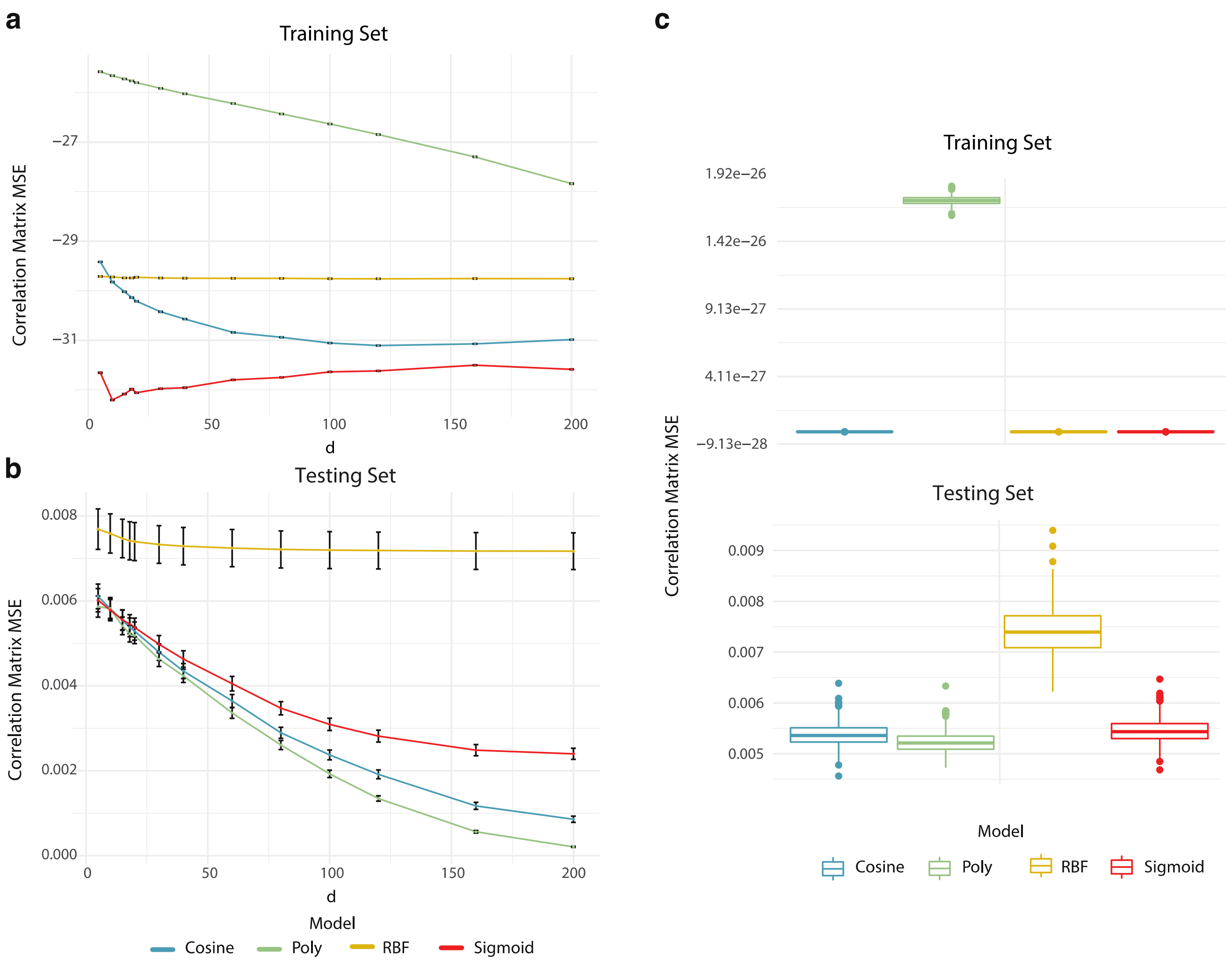

Supplementary Figure 3. KPCA model construction on the TwinsUK dataset. a,b, Training and test set metabolite correlation matrix reconstruction for a range of latent dimensionality values**.** Error bars correspond to one standard deviation from bootstrapping (*n* = 1,000 iterations). **c,** KPCA reconstruction correlation MSE for latent dimensionality *d* = 18 on training and test sets. The box represents the interquartile range (IQR), whiskers are up to 1.5x IQR, and plotted points are outliers.

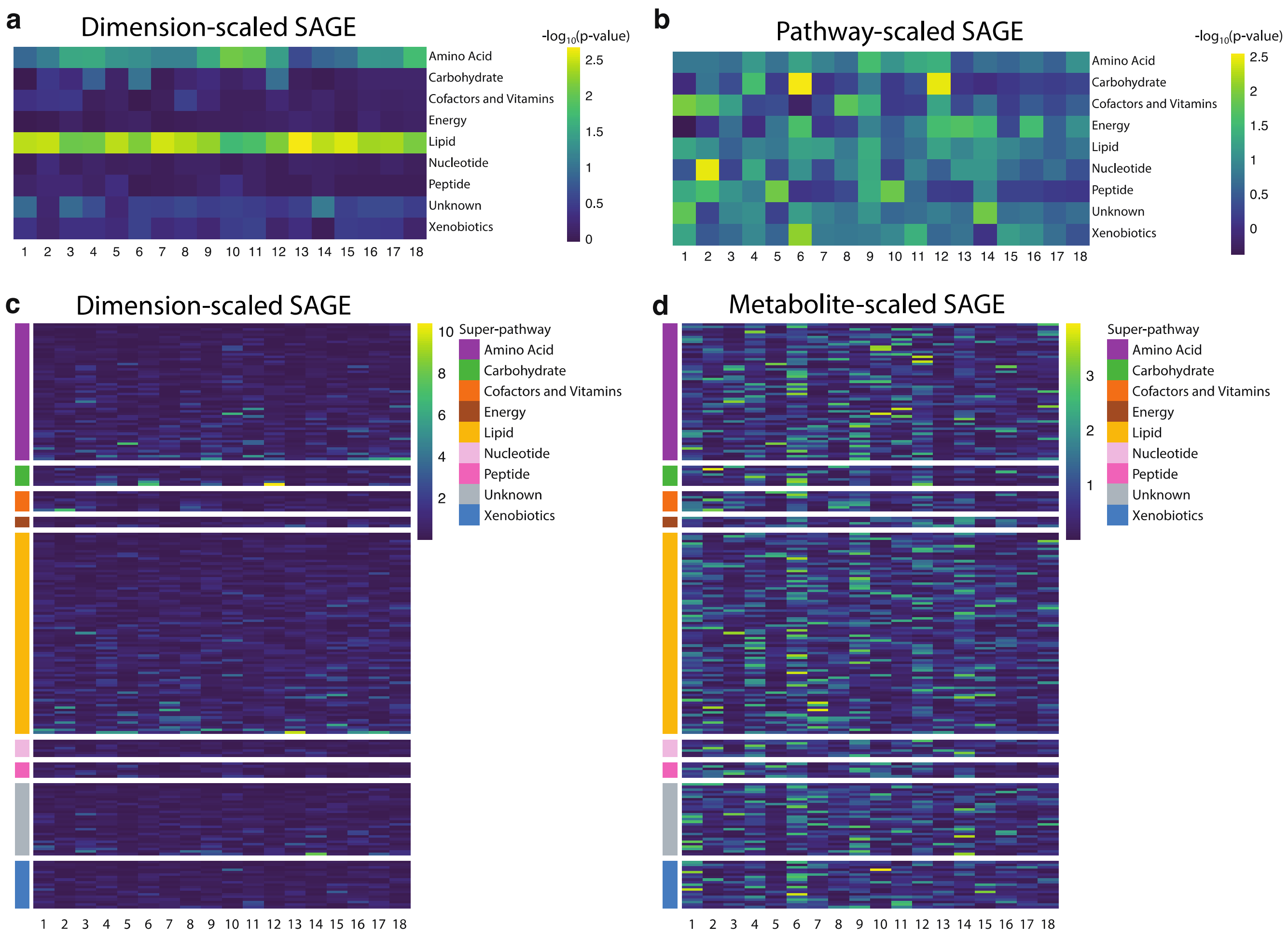

Supplementary Figure 4. Super-pathway and metabolite-level SAGE values for the VAE latent dimensions. **a**, SAGE values were scaled by dimension, i.e. set to standard deviation 1 for each column in the matrix. This highlights pathways that contributed the most to each dimension. Lipid and amino acid super-pathways showed the highest values for most dimensions, which can likely be attributed to the high number of metabolites in those pathways. **b**, SAGE values were scaled by pathway, i.e., set to standard deviation 1 for each row in the matrix. This highlights dimensions that contributed to a pathway the most. Taking into consideration the largest scaled SAGE values per pathway (red square marks), most super-pathways were represented by unique dimensions. **c**, Absolute metabolite SAGE were scaled by dimension, i.e., set to standard deviation 1 for each column in the matrix. This highlights metabolites that contributed the most to each dimension. Metabolites in the lipid and amino acid super-pathways showed the highest values for the majority of the dimensions. **d**, Absolute metabolite SAGE values were scaled by metabolite, i.e., set to standard deviation 1 for each row in the matrix. This highlights dimensions that contributed to a metabolite the most. Each dimension had a specific metabolic signature. The combination of these metabolites of a dimension outlined the distinct cellular mechanisms a dimension encodes.

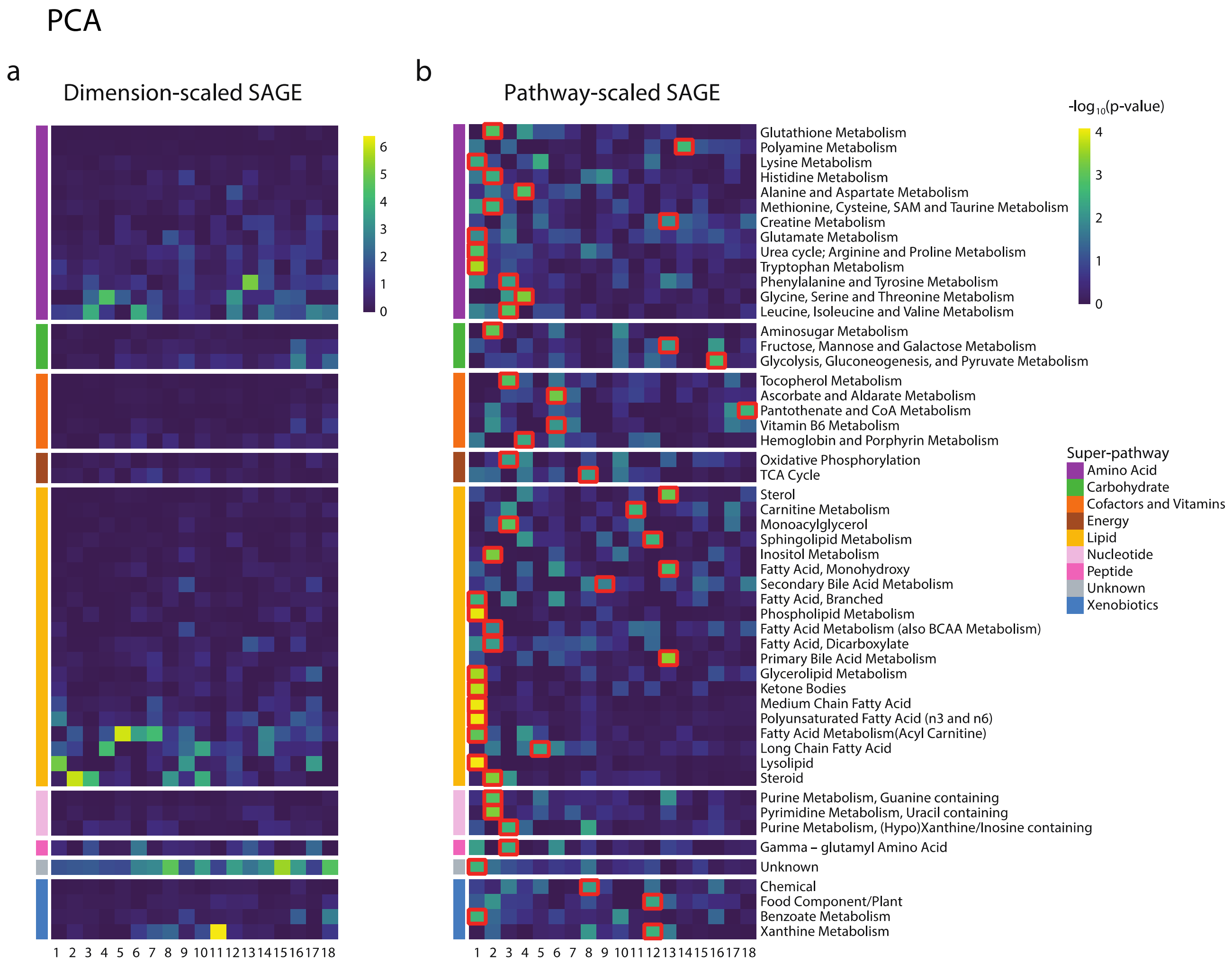

Supplementary Figure 5. Sub-pathway-level SAGE values for PCA latent dimensions. **a**, SAGE values were scaled by dimension, i.e., set to standard deviation 1 for each column in the matrix. This highlights pathways that contributed the most to each dimension. Lipid, unknown, and amino acid super-pathways showed the highest values for most dimensions, which can likely be attributed to the high number of metabolites in those pathways. **b**, SAGE values were scaled by pathway, i.e., set to standard deviation 1 for each row in the matrix. This highlights dimensions that contributed to a pathway the most. Taking into consideration the largest scaled SAGE values per pathway (red square marks), sub-pathways concentrated on the first 4 dimensions, especially on dimension 1. Other dimensions had primarily unrelated sub-pathways.

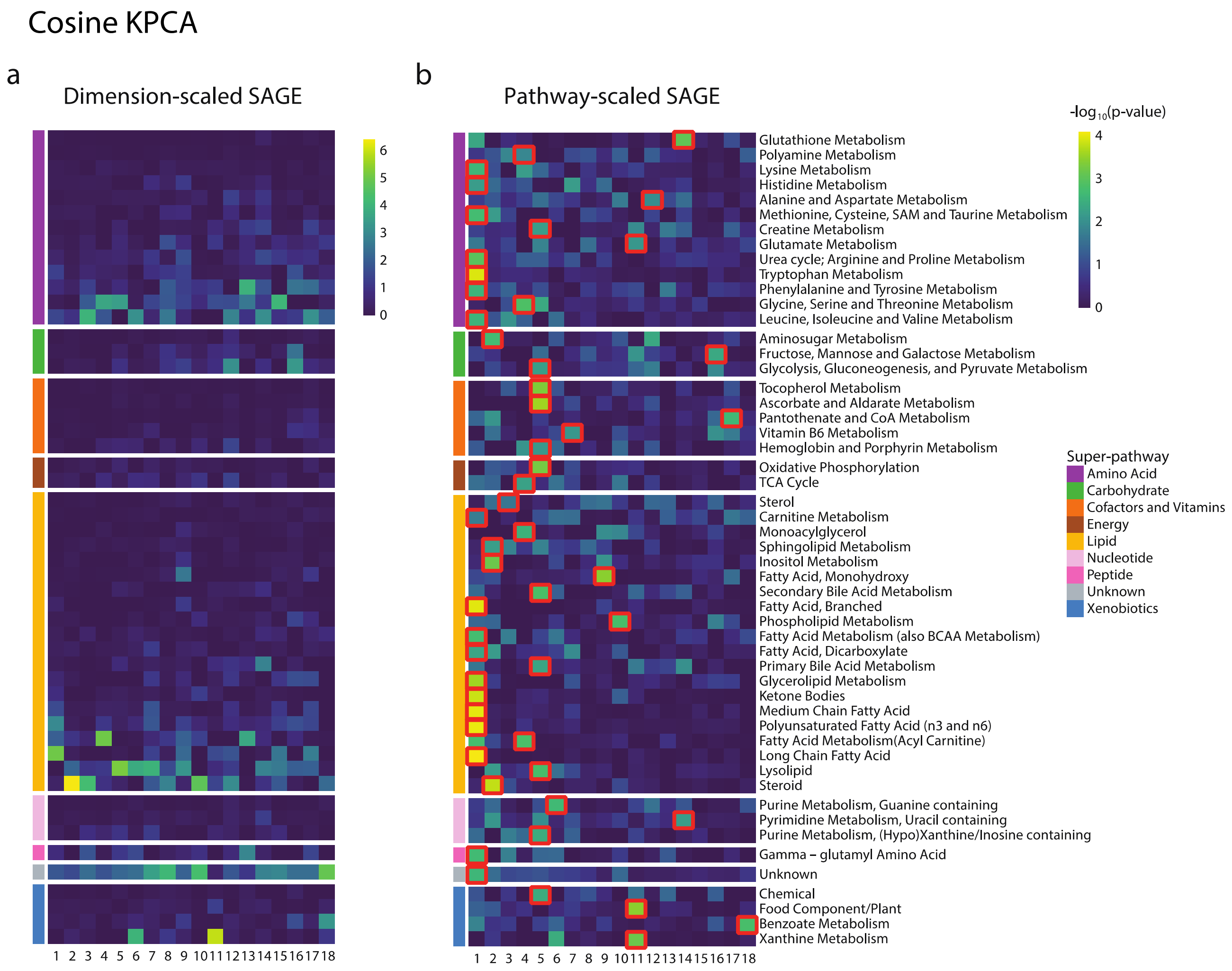

Supplementary Figure 6. Sub-pathway-level SAGE values for cosine-kernel KPCA latent dimensions. **A**, SAGE values were scaled by dimension, i.e., set to standard deviation 1 for each column in the matrix. This highlights pathways that contributed the most to each dimension. **b**, SAGE values were scaled by pathway, i.e., set to standard deviation 1 for each row in the matrix. This highlights dimensions that contributed to a pathway the most. Taking into consideration the largest scaled SAGE values per pathway (red square marks), sub-pathways concentrated on the first 5 dimensions, especially on dimension 1. Other dimensions had primarily unrelated sub-pathways.

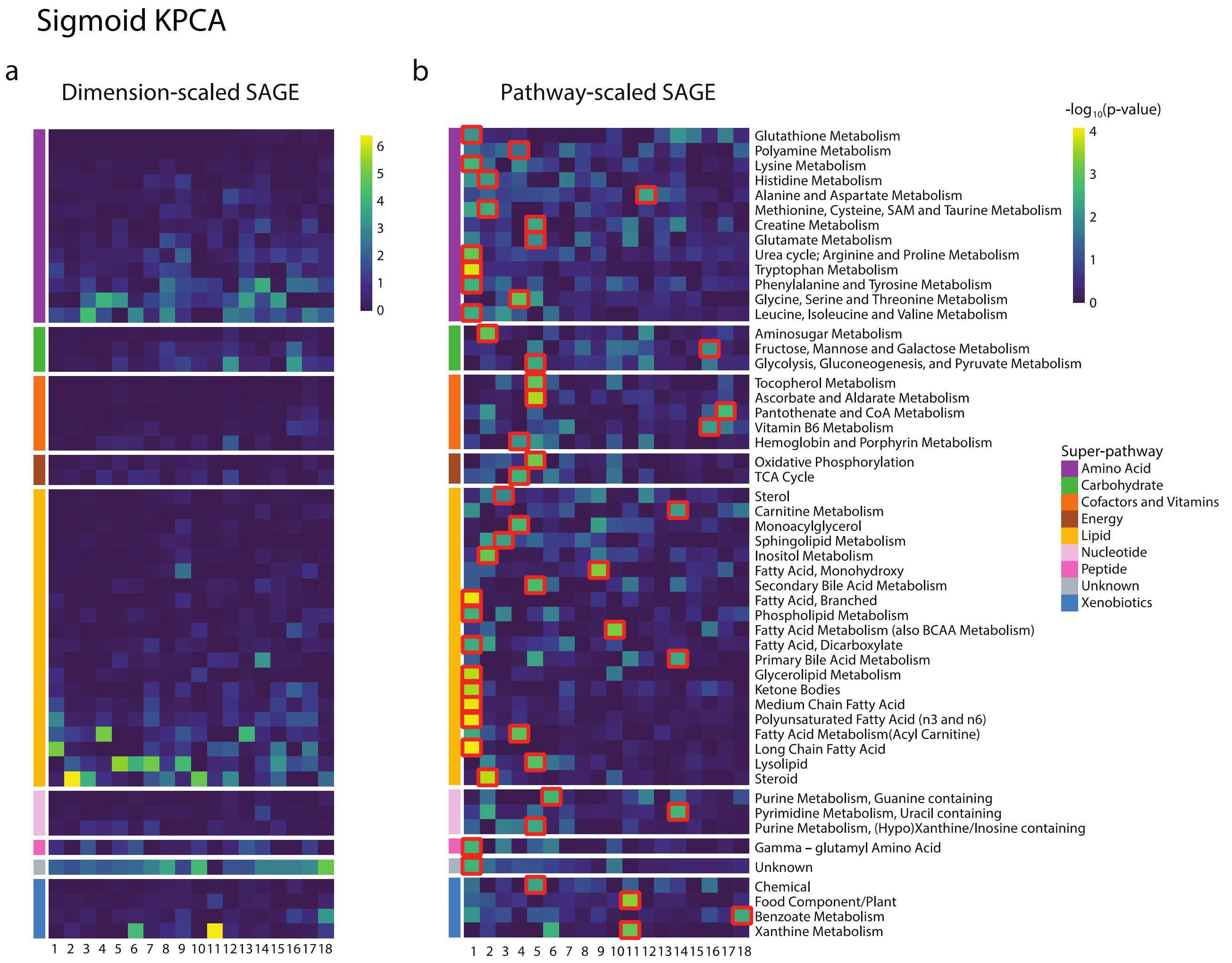

Supplementary Figure 7. Sub-pathway-level SAGE values for sigmoid-kernel KPCA latent dimensions. **a**, SAGE values were scaled by dimension, i.e., set to standard deviation 1 for each column in the matrix. **b**, SAGE values were scaled by pathway, i.e., set to standard deviation 1 for each row in the matrix. Taking into consideration the largest scaled SAGE values per pathway (red square marks), sub-pathways concentrated on the first 5 dimensions, especially on dimension 1. Other dimensions had primarily unrelated sub-pathways.

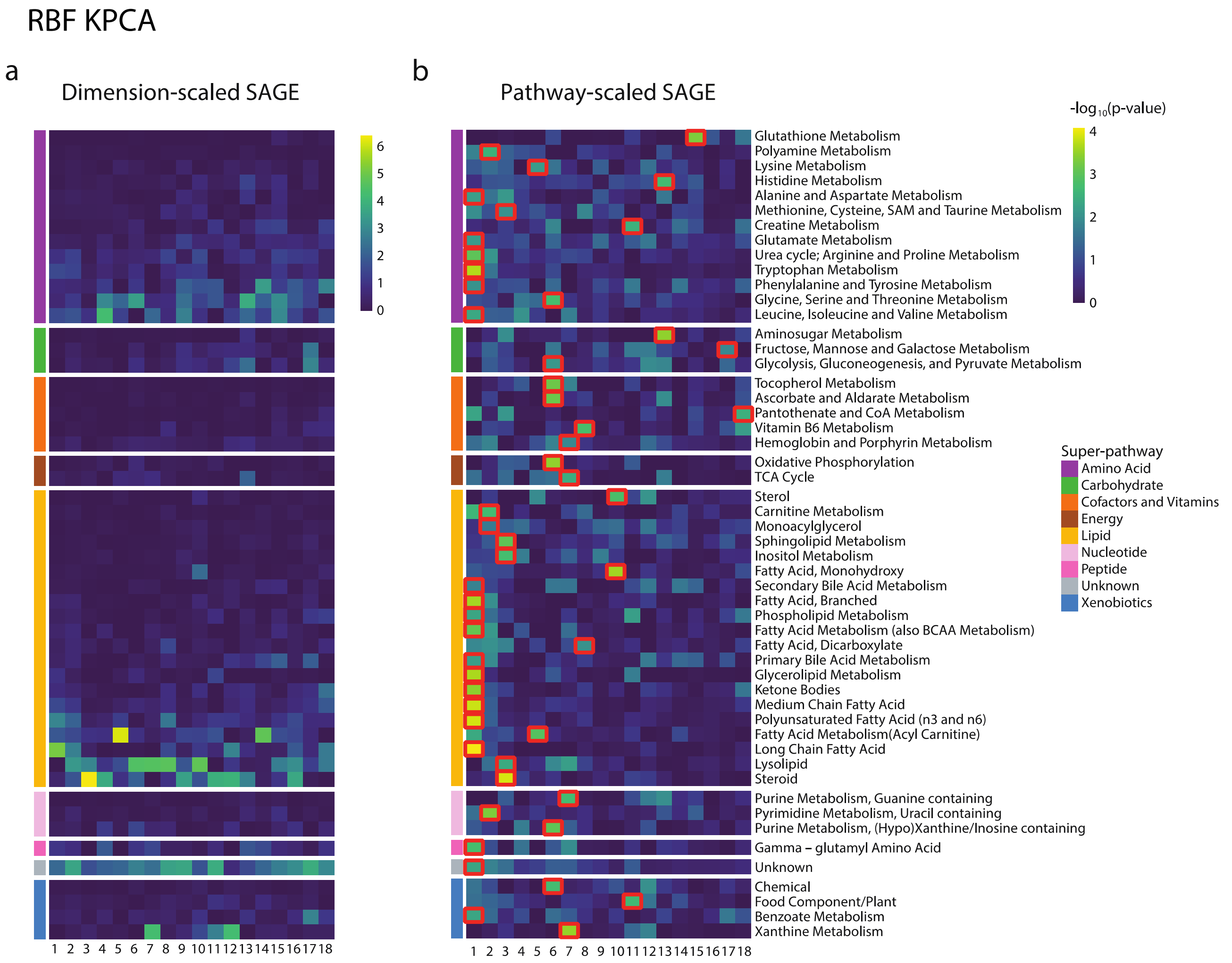

Supplementary Figure 8. Sub-pathway-level SAGE values for RBF-kernel KPCA latent dimensions. **a**, SAGE values were scaled by dimension, i.e., set to standard deviation 1 for each column in the matrix. **b**, SAGE values were scaled by pathway, i.e., set to standard deviation 1 for each row in the matrix. Taking into consideration the largest scaled SAGE values per pathway (red square marks), sub-pathways concentrated on the first 6 dimensions, especially on dimension 1, and, in contrast to other KPCA models, skipping dimension 4. Other dimensions had primarily unrelated sub-pathways.

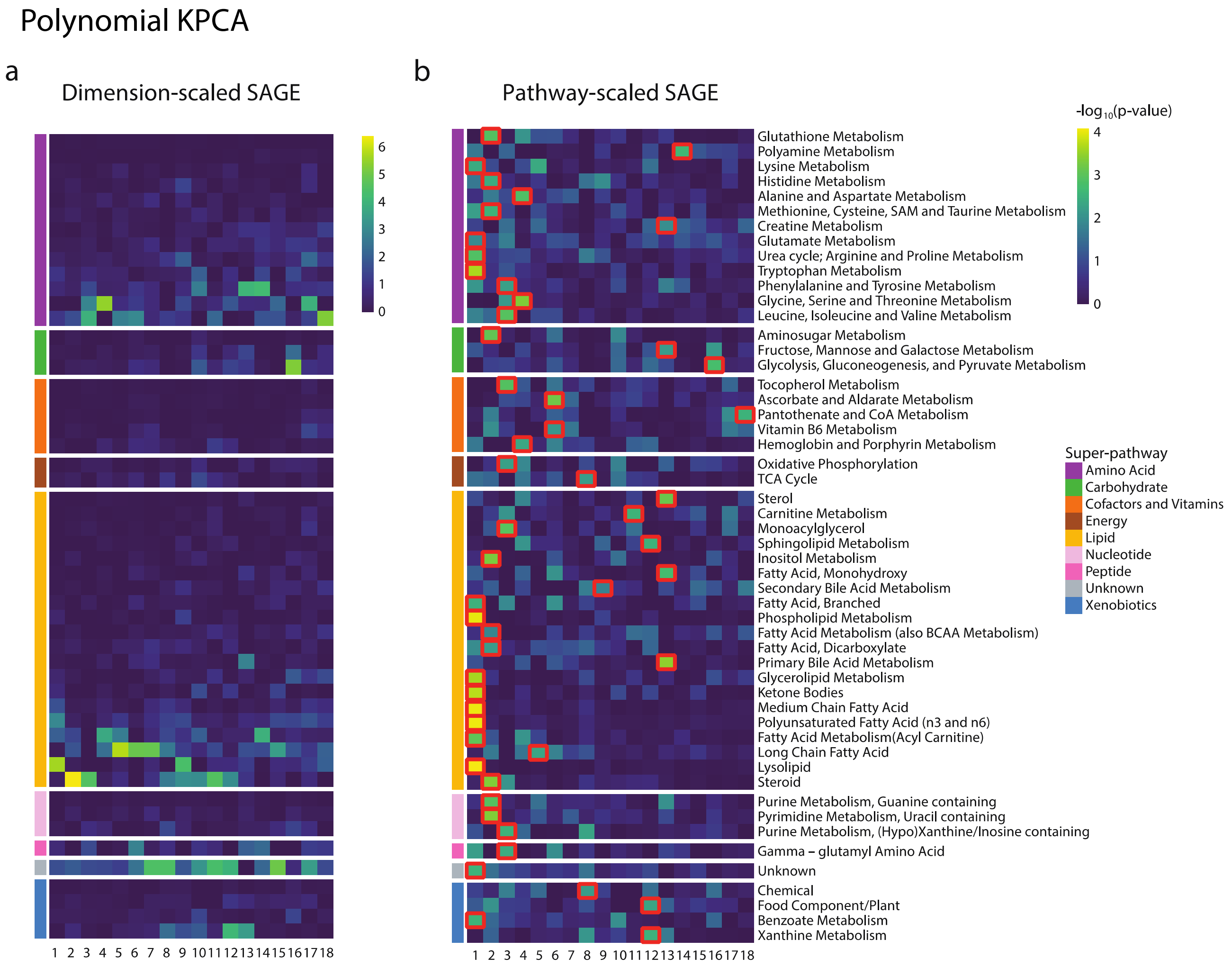

Supplementary Figure 9. Sub-pathway-level SAGE values for polynomial-kernel KPCA latent dimensions. **a**, SAGE values were scaled by dimension, i.e., set to standard deviation 1 for each column in the matrix. **b**, SAGE values were scaled by pathway, i.e., set to standard deviation 1 for each row in the matrix. Taking into consideration the largest scaled SAGE values per pathway (red square marks), sub-pathways concentrated on the first 4 dimensions, especially on dimension 1. Other dimensions had primarily unrelated sub-pathways.

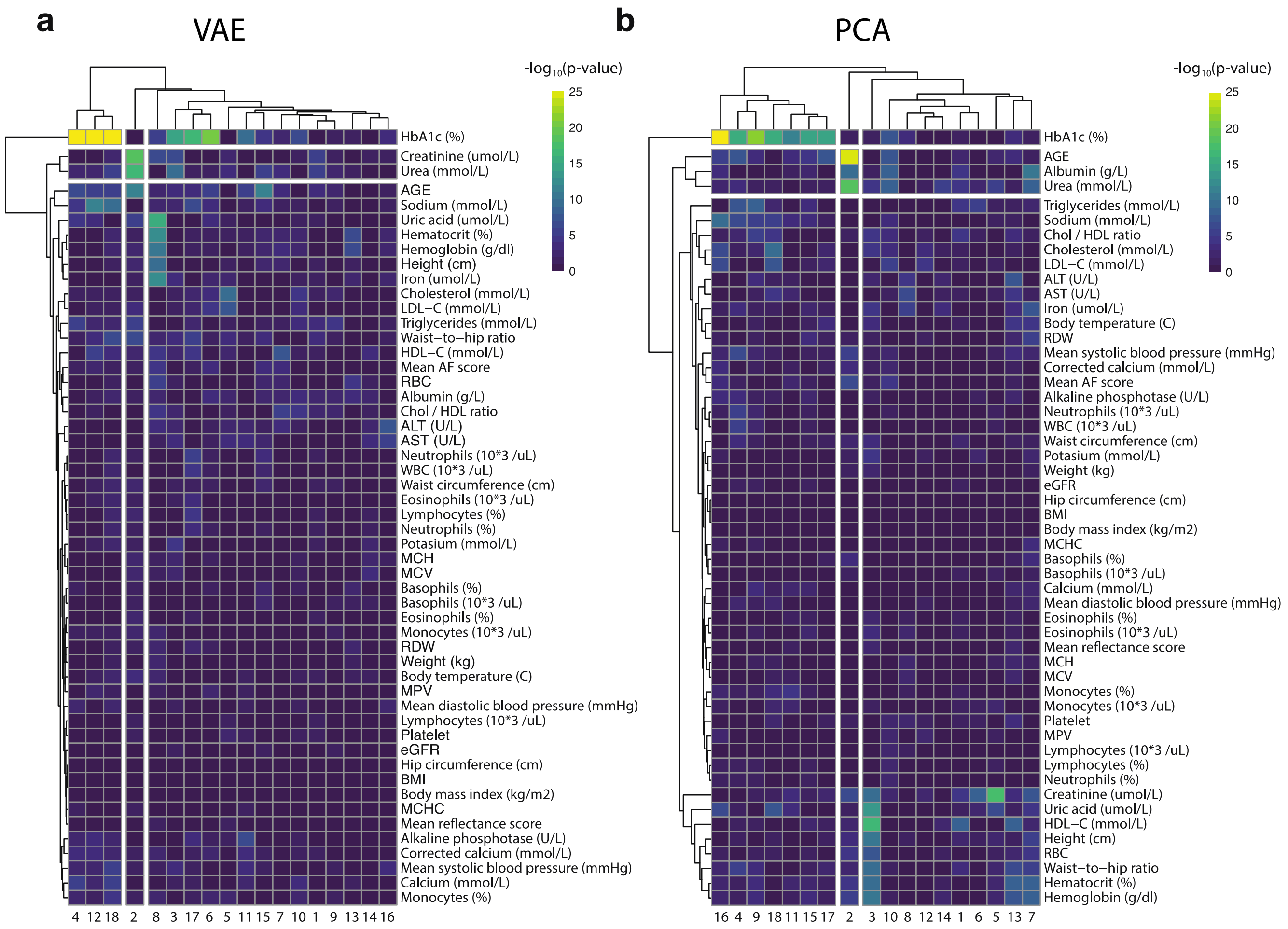

Supplementary Figure 10. Type 2 Diabetes clinical variable associations with VAE and PCA latent dimensions. Association heatmaps for **a**, VAE and **b**, PCA latent dimension values. Each latent dimension was associated with different combinations of clinical variables. VAE dimension 12 and PCA dimension 16 which correlated with QMDiab diabetes groups strongly associated with HbA1c (%) ($p = 6.2 \times 10^{-45}$ and $p = 1.1 \times 10^{-30}$ respectively, n = 358).

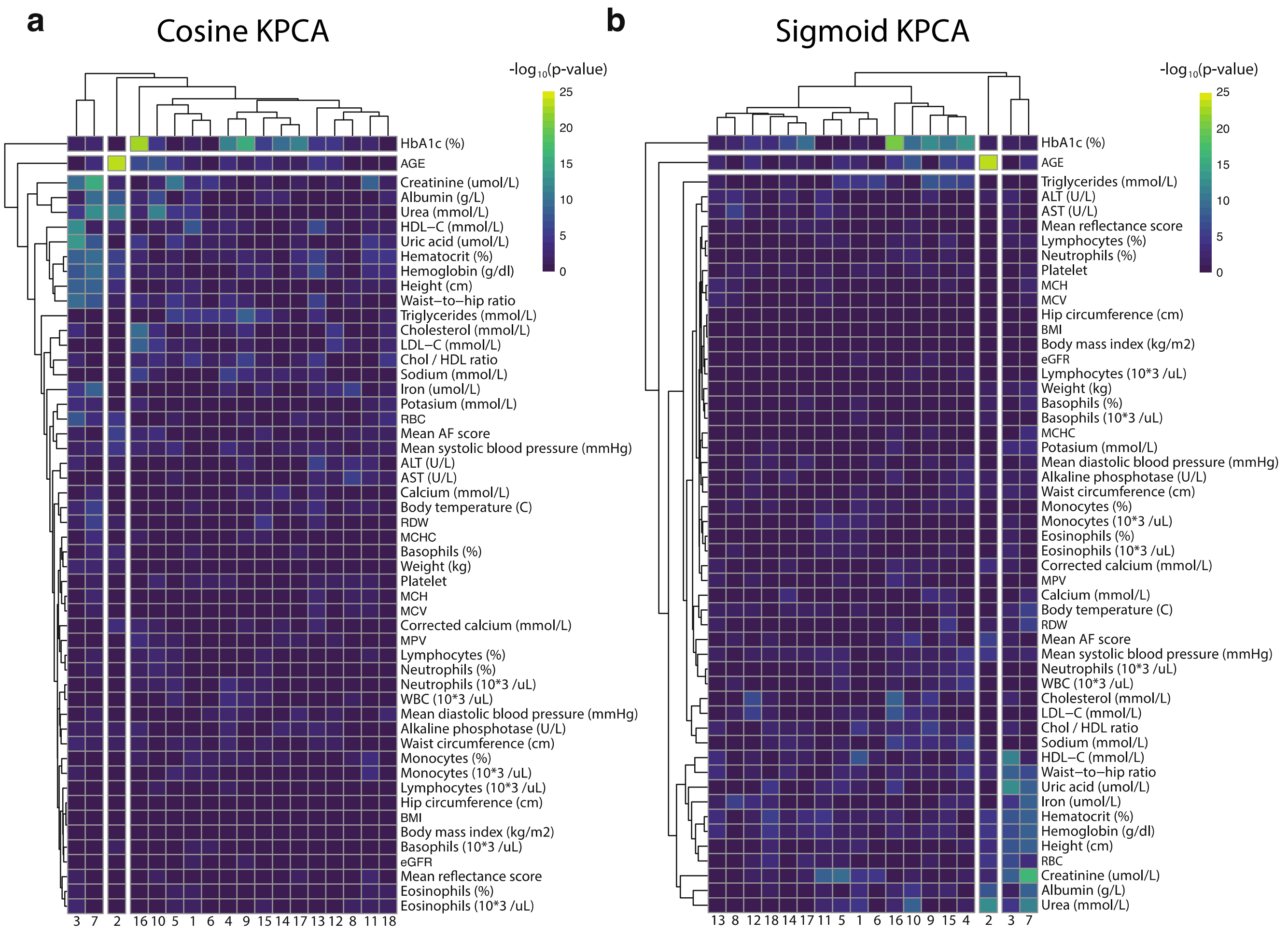

Supplementary Figure 11. Type 2 Diabetes clinical variable associations with cosine and sigmoid KPCA latent dimensions. Association heatmaps for **a**, cosine-kernel and **b**, sigmoid-kernel KPCA latent dimension values. Each latent dimension was associated with different combinations of clinical variables. Dimension 16 for both cosine and sigmoid KPCA strongly associated with HbA1c (%) ($p = 8.7 \times 10^{-23}$ and $p = 5.8 \times 10^{-21}$, respectively, $n = 358$).

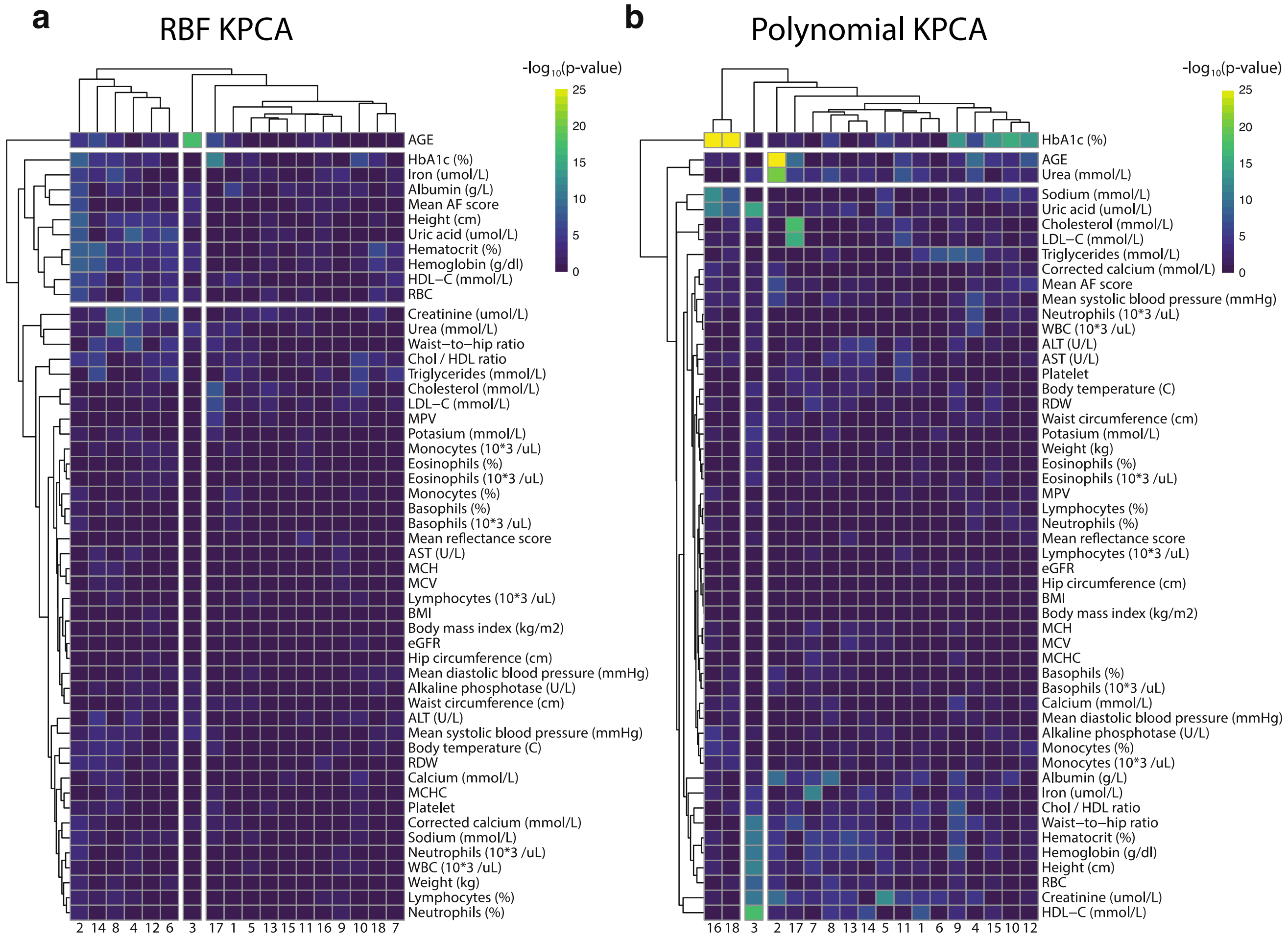

Supplementary Figure 12. Type 2 Diabetes clinical variable associations with RBF and polynomial KPCA latent dimensions. Association heatmaps for **a**, RBF-kernel and **b**, polynomial-kernel KPCA latent dimension values. Each latent dimension was associated with different combinations of clinical variables. RBF-KPCA dimension 17 and polynomial KPCA dimension 16 strongly associated with HbA1c (%) ($p = 1.1x10^{-12}$ and $p = 6.0x10^{-38}$, respectively, *n* = 358). The dimension with the strongest association with HbA1c of the KPCA models was dimension 16 of the polynomial transformed model.

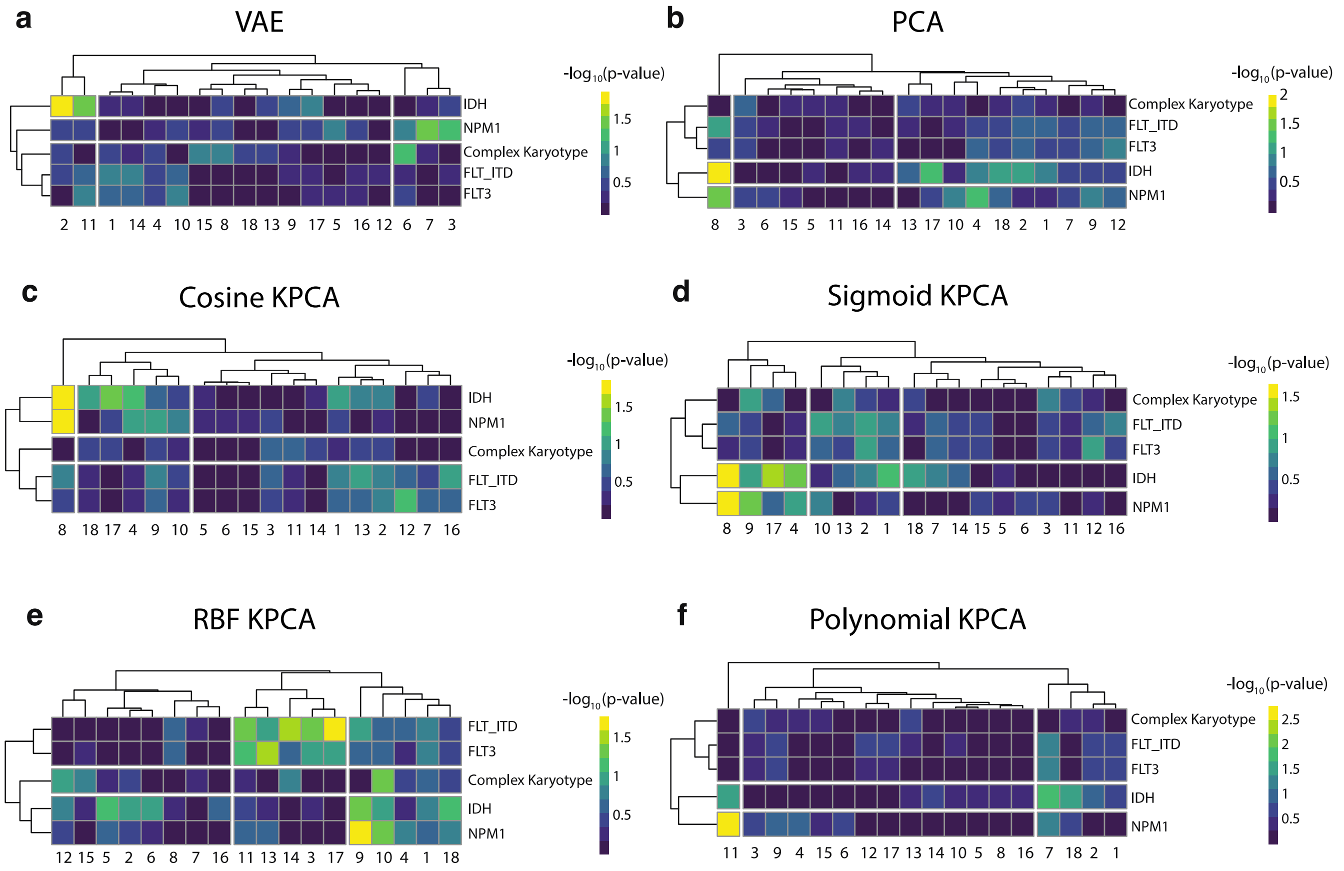

Supplementary Figure 13. AML mutation profile and latent dimension associations. Our dataset initially contained 21 AML-related mutations: *AML1-ETO, ASXL1, CBF, CEBPa, DNMT3A, EVI1, FLT-ITD, FLT3, IDH1, IDH2, KIT, KRAS, MLL, NPM1, NRAS, PHF6, PTEN, RUNX1, TET2, TP53, WT1*. To ensure adequate statistical power, we selected mutations with at least 10 samples per group, i.e., mutant or wildtype. This criterion retained 4 mutations and "complex karyotype" for our final statistical analysis. **a-f**, VAE, PCA, and all KPCA latent dimension association heatmaps. *IDH* and *NPM1* showed the strongest associations to the latent dimensions. WT = wildtype.

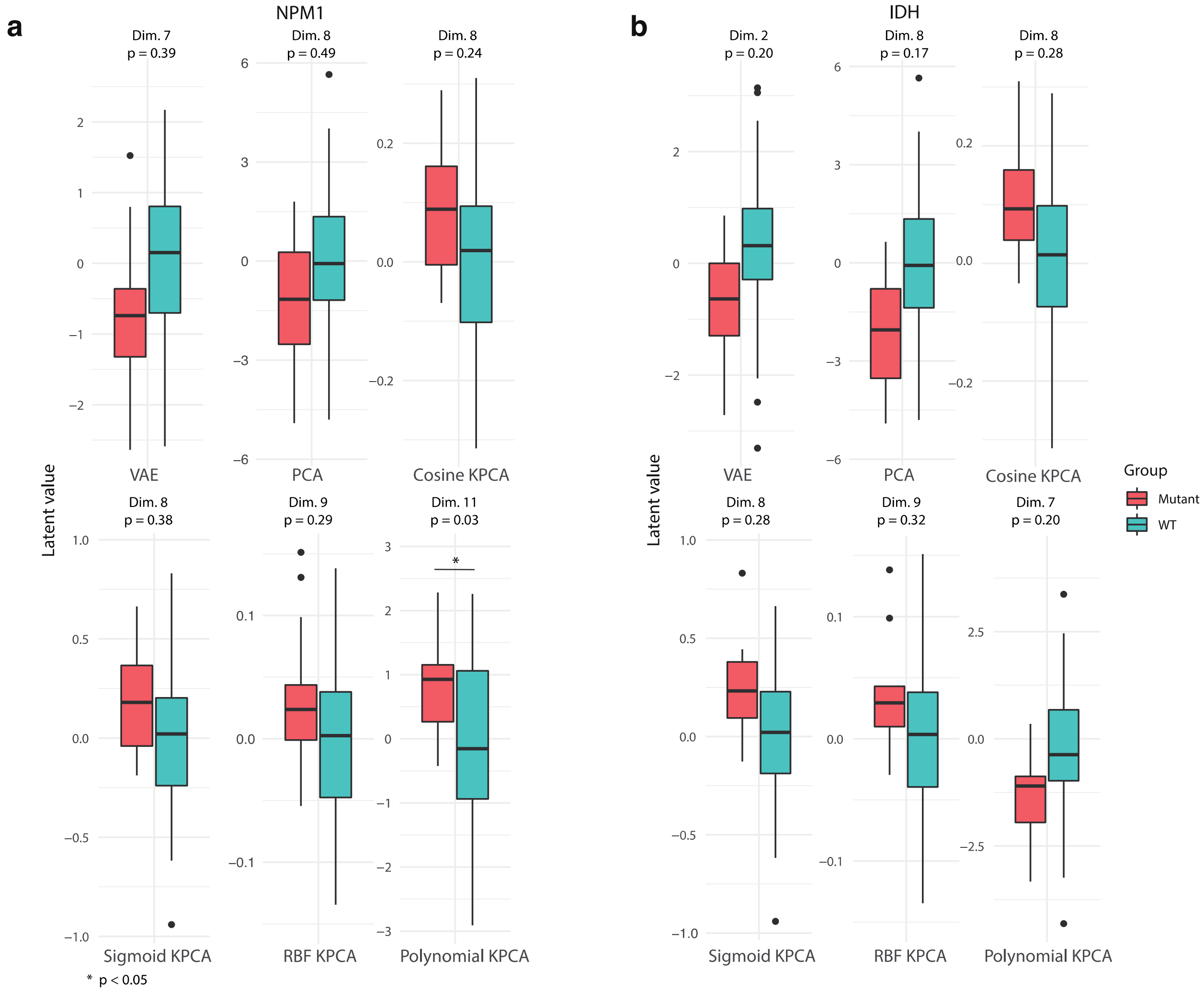

Supplementary Figure 14. AML mutation latent dimension association boxplots. **a**, Boxplot of model latent values for *NPM1* with FDR adjusted p-values (n = 85). Polynomial KPCA dimension 11 associated the strongest with *NPM1*. **b**, Boxplot of model latent values for *IDH*. PCA dimension 8 associated most with *IDH*. The box represents the interquartile range (IQR), whiskers are up to 1.5x IQR, and plotted points are outliers.

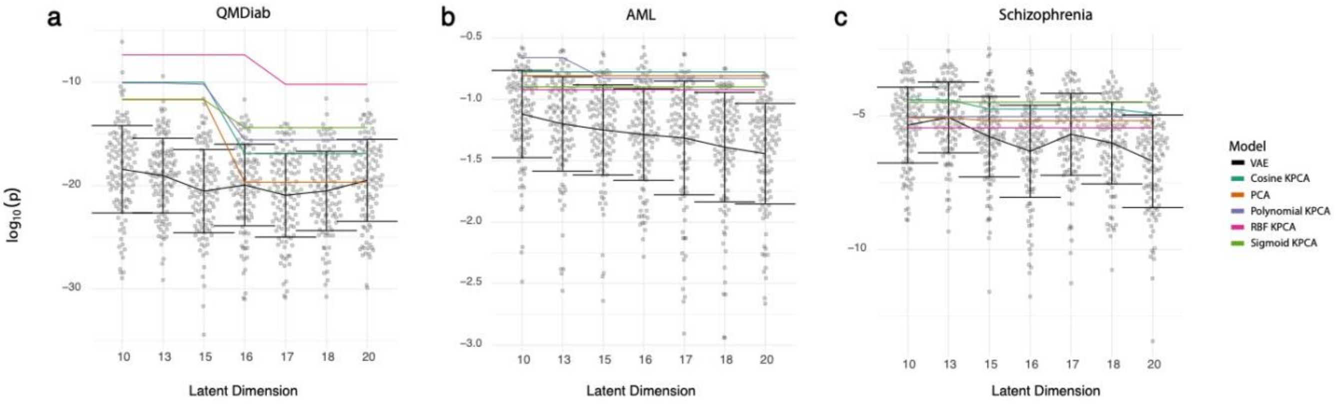

Supplementary Figure 15. Latent space associations with clinical outcomes for varying dimensions. For each latent dimension, *d* = 5, 10, 13, 15, 16, 17, 18, and 20, we calculated PCA and KPCA models and trained 100 VAE models. For each model, we calculated the $\log_{10}$ p-value of the strongest associated latent dimension with clinical outcomes in the **a,** type 2 diabetes (*n* = 358), **b,** AML (*n* = 85), and **c,** schizophrenia datasets (*n* = 201), respectively. Error bars on the VAE model correspond to one standard deviation from the 100 trained models.
