# Supplementary material for: Variational autoencoders learn transferrable representations of metabolomics data
Source: Commun Biol. 2022 Jun 30;5:645. doi: 10.1038/s42003-022-03579-3 (PMC9246987; doi:10.1038/s42003-022-03579-3)
Supplement: Supplementary file 2 — Description of Additional Supplementary Files [file 42003_2022_3579_MOESM2_ESM.pdf]

Description of Additional Supplementary Files

File name: Supplementary Data 1
Description: The source data behind the graphs 4d-f in this paper.

File name: Supplementary Data 2
Description: Nominal and FDR-adjusted pvalues of univariate associations between VAE latent dimensions and single metabolites for each of the disease datasets (Diabetes, Schizophrenia, and AML).
