# Supplementary material for: Variational autoencoders learn transferrable representations of metabolomics data
Source: Commun Biol. 2022 Jun 30;5:645. doi: 10.1038/s42003-022-03579-3 (PMC9246987; doi:10.1038/s42003-022-03579-3)
Supplement: Supplementary file 5 — Reporting Summary [file 42003_2022_3579_MOESM5_ESM.pdf]

nature research

Corresponding author(s): Jan Krumsiek

Last updated by author(s): May 9, 2022

Reporting Summary

Nature Research wishes to improve the reproducibility of the work that we publish. This form provides structure for consistency and transparency in reporting. For further information on Nature Research policies, see our Editorial Policies and the Editorial Policy Checklist.

Statistics

For all statistical analyses, confirm that the following items are present in the figure legend, table legend, main text, or Methods section.

n/a	Confirmed	
☐	☒	The exact sample size (n) for each experimental group/condition, given as a discrete number and unit of measurement
☐	☒	A statement on whether measurements were taken from distinct samples or whether the same sample was measured repeatedly
☐	☒	The statistical test(s) used AND whether they are one- or two-sided<br>*Only common tests should be described solely by name; describe more complex techniques in the Methods section.*
☐	☒	A description of all covariates tested
☐	☒	A description of any assumptions or corrections, such as tests of normality and adjustment for multiple comparisons
☐	☒	A full description of the statistical parameters including central tendency (e.g. means) or other basic estimates (e.g. regression coefficient) AND variation (e.g. standard deviation) or associated estimates of uncertainty (e.g. confidence intervals)
☐	☒	For null hypothesis testing, the test statistic (e.g. F, t, r) with confidence intervals, effect sizes, degrees of freedom and P value noted<br>*Give P values as exact values whenever suitable.*
☒	☐	For Bayesian analysis, information on the choice of priors and Markov chain Monte Carlo settings
☒	☐	For hierarchical and complex designs, identification of the appropriate level for tests and full reporting of outcomes
☒	☐	Estimates of effect sizes (e.g. Cohen's d, Pearson's r), indicating how they were calculated

Our web collection on statistics for biologists contains articles on many of the points above.

Software and code

Policy information about availability of computer code

Data collection	No software was used to collect data in this study.
Data analysis	Scripts to train our variational autoencoder model, tune hyperparameters, assess performance, and reproduce figures can all be found at the GitHub repository https://github.com/krumsieklab/mtVAE

For manuscripts utilizing custom algorithms or software that are central to the research but not yet described in published literature, software must be made available to editors and reviewers. We strongly encourage code deposition in a community repository (e.g. GitHub). See the Nature Research guidelines for submitting code & software for further information.

Data

Policy information about availability of data

All manuscripts must include a data availability statement. This statement should provide the following information, where applicable:
- Accession codes, unique identifiers, or web links for publicly available datasets
- A list of figures that have associated raw data
- A description of any restrictions on data availability

All data has been deposited at https://figshare.com/s/6716415ce4b4e8295f5b
Note that this has to be a private repository until the time of publication.

Field-specific reporting

Please select the one below that is the best fit for your research. If you are not sure, read the appropriate sections before making your selection.

☒ Life sciences ☐ Behavioural & social sciences ☐ Ecological, evolutionary & environmental sciences

For a reference copy of the document with all sections, see nature.com/documents/nr-reporting-summary-flat.pdf

Life sciences study design

All studies must disclose on these points even when the disclosure is negative.

Sample size	For the TwinsUK dataset, our sample size is 4,644 individuals. Deep learning methodologies (including those used in this manuscript) generally require thousands of samples for training. As our model was trained on this set, we believe this sample size to be sufficient. For our validation datasets, the QMDiab study cohort consists of 358 subjects, the Schizophrenia cohort consists of 226 subjects, and the Acute Myeloid Leukemia (AML) cohort consists of 86 individuals. All of these sample sizes reflect the number of individuals for which metabolomics measurements were available in their respective studies, minus the exclusions listed below.
Data exclusions	Samples which were missing values for over 30% of measured metabolites were excluded. Similarly, metabolites with more than 10% of missing samples were also removed.
Replication	Using the trained model provided in the GitHub repository, all results have been reproduced. Due to randomness introduced when the model is trained, when the model is created from scratch, certain result plots will not replicate exactly, but the key findings persist.
Randomization	We did not conduct the studies to collect our data. For details on randomization, please refer to the original designs of the TwinsUK, QMDiab, Schizophrenia, and AML studies in the Methods section of our manuscript.
Blinding	We did not conduct the studies to collect our data. For details on blinding, please refer to the original designs of the TwinsUK, QMDiab, Schizophrenia, and AML studies in the Methods section of our manuscript.

Reporting for specific materials, systems and methods

We require information from authors about some types of materials, experimental systems and methods used in many studies. Here, indicate whether each material, system or method listed is relevant to your study. If you are not sure if a list item applies to your research, read the appropriate section before selecting a response.

Materials & experimental systems

n/a	Involved in the study	
☒	☐	Antibodies
☒	☐	Eukaryotic cell lines
☒	☐	Palaeontology and archaeology
☒	☐	Animals and other organisms
☐	☒	Human research participants
☐	☒	Clinical data
☒	☐	Dual use research of concern

Methods

n/a	Involved in the study	
☒	☐	ChIP-seq
☒	☐	Flow cytometry
☒	☐	MRI-based neuroimaging

Human research participants

Policy information about studies involving human research participants

Population characteristics	For our study, we included data from 4,644 subjects from the TwinsUK registry (4,256 females, 388 males), ranging in age from 18 to 103 years old. From the QMDiab study, we included measurements from 358 subjects (176 females, 182 males; 188 diabetic, 177 non-diabetic) between the ages of 23 and 71, predominantly of Arab, South Asian, and Filipino descent. For the schizophrenia study, we included measurements from 207 subjects (84 females, 142 males; 102 schizophrenic, 105 non-schizophrenic) between the ages of 18 and 65, predominantly of Arab descent. For the AML cohort of patients, we include the measurements of 85 subjects of which 43 responded to therapy and 42 did not (34 females, 51 males) between the ages of 17 and 60.
Recruitment	We did not conduct the studies to collect our data. For details on subject recruitment, please refer to the original designs of the TwinsUK, QMDiab, Schizophrenia, and AML studies in the Methods section of our manuscript.
Ethics oversight	TwinsUK: Ethical approval was granted by the St Thomas' Hospital ethics committee.<br>QMDiab: The study was approved by the Institutional Review Boards of the Hamad Medical Corporation (HMC) and Weill Cornell Medicine-Qatar (WCM-Q).<br>Schizophrenia Cohort: Approval for the study was obtained from the HMC and WCM-Q Institutional Review Boards.

AML cohort: The study was approved by the institutional review board at the National Cancer Institute and each of the study centers.

Note that full information on the approval of the study protocol must also be provided in the manuscript.

Clinical data

Policy information about clinical studies

All manuscripts should comply with the ICMJE guidelines for publication of clinical research and a completed CONSORT checklist must be included with all submissions.

Clinical trial registration	NCT00049517
Study protocol	https://clinicaltrials.gov/ct2/show/NCT00049517
Data collection	We did not conduct this clinical study, we only used the resulting data in our analysis. For details on collection and recruitment, please refer to the study protocol above.
Outcomes	We did not conduct this clinical study, we only used the resulting data in our analysis. For details on outcome measurement and assessment, please refer to the study protocol above.
